# Toxicities of CAR T-cell therapy: a review of current literature

**DOI:** 10.1097/MS9.0000000000001375

**Published:** 2023-10-20

**Authors:** Muhammad Sohaib Asghar, Syed M. Ismail Shah, Anooja Rani, Sana Kazmi, Ilma S. Savul, Janta Ukrani, Farmanullah Khan, Chaudhary A. Hasan, Navin Rathore, Maria Syed, Shiwani Keswani, FNU Surkasha, Doongro Mal, Dileep Kumar

**Affiliations:** a Department of Internal Medicine, Dow Medical College, Dow University of Health Sciences; b Department of Internal Medicine, Ziauddin Medical University; c Department of Medicine, Liaquat University of Medical and Health Sciences; d Department of Medicine, Ghulam Muhammad Mahar Medical College, Sukkur; e Department of Medicine, Liaquat National Hospital and Medical College; f Department of Surgery, Aga Khan University Hospital, Karachi, Pakistan; g Department of Internal Medicine, Mather Hospital-Northwell Health, New York; h Department of Internal Medicine, St. Joseph Medical Center, Houston; i Division of Nephrology and Hypertension, Mayo Clinic-Rochester, Minnesota, USA

**Keywords:** CAR T-cell therapy, chimeric antigen receptor, cytokine release syndrome (CRS), neurological toxicity, neurology, pharmacology

## Abstract

The chimeric antigen receptor (CAR) design, first invented by Zelig Eshhar, paved the way for the use of genetically modified T-cells in targeted therapy against cancer cells. Since then, it has gone through many generations, especially with the integration of co-stimulation in the second and third-generation CARs. However, it also mounts a hyperactive immune response named as cytokine release syndrome with the release of several cytokines eventually resulting in multiple end-organ toxicities. The severity of cytokine release syndrome depends upon certain factors such as the tumor burden, choice of co-stimulation, and degree of lymphodepletion, and can manifest as pulmonary edema, vascular leak, renal dysfunction, cardiac problems, hepatic failure, and coagulopathy. Many grading criteria have been used to define these clinical manifestations but they lack harmonization. Neurotoxicity has also been significantly associated with CAR T-cell therapy but it has not been studied much in previous literature. This review aims to provide a comprehensive account of the clinical manifestations, diagnosis, management, and treatment of CAR T-cell associated neurotoxicity.

## Introduction

Highlights

- Hyperactive immune response in chimeric antigen receptor-based therapy can lead to neurotoxicity.
- This has also been significantly underreported in previous literature.
- This review aims to provide a comprehensive account of the treatment of chimeric antigen receptor T-cell associated neurotoxicity.

The usage of targeted adoptive cell therapy is aimed at using the body’s immune system against tumor cells. The use of chimeric antigen receptor (CAR) in genetically modified T-cells was first invented by Zelig Eshhar; however, there are some ambiguities about this fact. It paved the way for allogeneic or autologous T-cells to be genetically modified using CAR, providing therapy for cancer patients. The first successful cancer treatment using CAR T-cell therapy was performed in 2010 for an advanced follicular lymphoma patient. Since then, it has gone through five generations and undergone multiple upgrades to provide a more sophisticated treatment^1^. Since then, it has been used in B-cell acute lymphoblastic leukemia (ALL), B-cell non-Hodgkin lymphoma, including diffuse large B-cell lymphoma (DLBCL), follicular lymphoma with (DLBCL), high-grade B-cell lymphoma, primary mediastinal large B-cell lymphoma, and mantle cell lymphoma, and more recently in targeted therapy of multiple myeloma. However, it also develops an overwhelming immune response in some patients in the form of cytokine release syndrome (CRS). Clinically, this is seen as fever, low blood pressure, respiratory distress, and multiple organ failure amongst other manifestations. As a result of on/off-tumor toxicity, antigens targeted by CAR T-cells can destroy normal B-cells apart from cancerous ones. Therefore, B-cell aplasia can result^2^, which is also an indicator of the CAR T-cell activity. Neurotoxicity is another common pathology associated with CAR T-cell therapy in the clinical setting. Neurologic toxicity is considered separate from CRS even if certain common cytokines are involved in its development. It is still early days for targeted CAR T-cell therapies but studies have shown promising results with cancer remissions right after treatment and a cancer-free period of more than 12 months reported in the literature. Future and ongoing research indicates that there can be more insights of role of CAR T-cell therapy in treating solid organ tumors like breast cancer, lung cancer, and possibly brain cancer. Other researchers are extensively finding ways to reduce therapy side effects and investigating the ways to extend the length of time that CAR T-cells might contain cancer.

In this review, we present a detailed discussion of the pathophysiology, clinical manifestations, diagnosis, and management of the neurological risk profile in patients receiving CAR T-cell therapy.

## Methods

An extensive literature was carried out using PUBMED/MEDLINE, Scopus, Web of Science (WoS), and Google Scholar from its inception to June 2020. The following search string was employed: (‘CAR T-cell therapy’) AND (‘neurotoxicity’) AND (‘pathophysiology’ OR ‘clinical manifestations’ OR ‘diagnosis’ OR ‘management’ OR ‘treatment’). All articles in a language other than the English were excluded from our review. We tried to formulate this narrative review by including relevant text and knowledge from prior literature.

## Overview of CAR T-cell therapy

CARs commonly contain three modules^3^. They are all in series, namely: an antigen recognition domain, a transmembrane element, and a signaling endodomain. The first generation of CARs contained a single-chain variable fragment, coexpressing elements from a monoclonal antibody such as the antigen-binding proteins, with the CD3ζ endodomain of the TCR/CD3 complex. However, they failed to show the required T-cell expansion and persistence^4^. The invention of second-generation and third-generation CARs came with the integration of one or two co-stimulatory domains, respectively. CD28 or 4-1BB signaling elements are known to be the best known and widely tested co-stimulatory domains. Co-stimulation prevents the unresponsive state seen in primary TCR stimulation, known as ‘anergy’. CAR T-cell therapy determines the target specificity and affinity, similar to the light chain region of an antibody. It does so without the need for histocompatibility complex activation, which imparts more flexibility to it. This is particularly useful in targeting tumor cells with down-regulated HLA expression and proteasomal antigen processing^5^. CAR T-cell therapy is important for T-cell expansion and persistence. Another important advantage is its ability to bind to protein as well as glycolipid and carbohydrate structures. It can be active in both CD4+ and CD8+ cells and there is only a minimal risk of autoimmunity and graft-versus-host disease. Other than that, role of different chemokines (GM-CSF, CXCL8, etc.) have a more comprehensive pathophysiology that contributes to ICANS.

## Clinical manifestations and pathophysiology of CRS

### Clinical manifestations

CRS is an acute systemic inflammatory response syndrome caused by the release of inflammatory cytokines such as Interleukin-2 (IL-2), IL-2 receptor a, IL-6, IL-8, IL-10, interferon-γ, and tumor necrosis factor. It varies from being self-limiting to being treated in an ICU^6^. The first symptom observed in CRS is fever^7–9^. According to the clinical trials^7,10^, the onset and the duration of the fever varied with the grading of CRS. Patients with a grade greater than 4, experienced fever within 25 h whereas patients with a grade less than 3, experienced fever after 12 days of the CAR T-cell infusion. In addition to fever, the patients also experience tachycardia, hypotension, hypoxia, and some neurological changes such as decreased attention span, language disturbance, and impaired handwriting. Some of the severe neurological manifestations include obtundation, seizures, and cerebral edema^11,12^.

The severity of CRS is determined by the elevation of IL-6, IFN-Y, and soluble IL2Ra serum markers, which show a marked increase in severe CRS as compared to the CRS without severity^13^. Severe CRS manifests as pulmonary edema, vascular leak, renal dysfunction, cardiac problems, hepatic failure, and coagulopathy^9^. According to a phase 1 trial at Memorial Sloan Kettering Cancer Center (MSKCC)^14^, the severity of CRS is associated with a higher disease burden as compared to a lower disease burden. 41% of the patients with high disease burden were observed to have severe CRS, whereas only 5% of patients with lower disease burden experienced it.

Multiple end-organ toxicities caused by the CAR T-cell infusion are mostly reversible. The constitutional symptoms include fever, malaise, fatigue, and headache. CRS has an impact on the human heart leading to cardiac problems like QT-prolongation^6^, troponinemia^15^, arrhythmias including sinus tachycardia^15–17^, and decreased left ventricular ejection fraction^6,15,17^. It also causes hepatic impairment by increasing the hepatic enzymes and bilirubin as observed in the clinical trials conducted in 2012^18^ and 2016^19^. Similarly, there is an increase in the serum creatinine level, which suggests renal insufficiency^6,18^ which further leads to hypokalemia, hyponatremia, and hypophosphatemia. Tumor lysis syndrome^20^ and muscle damage^6,19^ has also been reported as an effect of CRS. Furthermore, there are respiratory problems following the CAR T-cell infusion and they include dyspnea, increased respiratory rate, and pleural effusions. Hematologic toxicities have also been observed in some reports^6,15^ which show that there is a development of anemia, neutropenia, and thrombocytopenia where conditioning chemotherapy regimens have been used. An increased prothrombin time, partial thromboplastin time, and decreased fibrinogen levels have also been seen in a clinical trial^21^. In some cases^8^, disseminated intravascular coagulation may be the consequence of the hematological toxicity.

### Pathophysiology

CAR T-cells target antigens, proliferate, and become activated to secrete large amounts of cytokines such as IL-1, IL-6, IL-8, IL-10, IL-12, TNF-α, IFN-γ, MCP-1, and MIP-1α. Immune cells stimulated include lymphocytes such as B-cells, T-cells and natural killer cells, and/or myeloid cells including macrophages, dendritic cells, and monocytes. Uninterrupted stimulation of the immune system, particularly macrophages, can explain the development of hemophagocytic lymphohistiocytosis/macrophage activation syndrome. Again, some cytokines are involved^13^ and a genetic predisposition also exists in these patients. Moreover, IL-6 is an important cytokine of CRS, highly associated with macrophages^22^, which initiates a proinflammatory IL-6 mediated signaling cascade^9^. The severity of CRS depends upon the tumor burden. The choice of co-stimulatory ligand and the level of lymphodepletion, both of which are associated with enhanced T-cell proliferation, are also known to affect the severity of CRS. Endothelial activation is also involved. High serum concentrations of VWF, Ang2, and endothelium-activating cytokines, such as IL-6 and interferon-γ, can explain the capillary leak and coagulopathy associated with severe CRS^7^.

### Grading of CRS

The Common Terminology Criteria for Adverse Events (CTCAE) 4.3 (Table 1)^24^ was the first grading scheme used, which was modified by later clinical trials (Table 2). Lee *et al.* and others redefined the clinical presentations associated with CRS grading in CTCAE 4.3. Guidelines were altered with regards to hypoxia requiring oxygen support, hypotension, and responsiveness to vasopressors and other end-organ toxicities, particularly in grades 2 and 3. The CARTOX consensus group defined hypotension in their criteria as systolic blood pressure less than 90 mmHg in adults^25^. Interestingly, other symptoms of CRS were not included in the grading criteria because they were always associated with hypotension and/or hypoxia. It is evident from the grading criteria in Tables 1 and 2 that disparities exist in the guidelines. Hypoxia and hypotension have not been consistently defined as well. Therefore, ASTCT, the consensus grading system was formulated in 2018, which called for the harmonization of CRS grading and definitions^23^.

**Table 1:** Comparison of cytokine release syndrome grading using CTCAE versions 4.03 and 5.0^23^.

	Grade 1	Grade 2	Grade 3	Grade 4	Grade 5
Version 4.03^24^	Mild reaction; infusion interruption not indicated; intervention not indicated	Therapy or infusion interruption indicated, but responds promptly to symptomatic treatment (antihistamines, NSAIDS, narcotics, IV fluids); prophylactic medications indicated for ≤24 h	Prolonged (e.g. not rapidly responsive to symptomatic medication and/or brief interruption of infusion); recurrence of symptoms following initial improvement; hospitalization indicated for clinical sequel (such as renal impairment, pulmonary infiltrate)	Life-threatening consequences; pressor or ventilator support indicated	Death
Version 5.0^25^	Fever, with or without constitutional symptoms	Hypotension responding to fluids. Hypoxia responding to <40% FiO2	Hypotension is managed with one pressor. Hypoxia requiring ≥40% FiO2	Life-threatening consequences; urgent intervention needed	Death

Lee *et al.* 2019^23^. Table is adapted from above publication.

**Table 2:** Comparison of grading criteria utilized in different clinical trials^23^.

	Lee *et al*. criteria^9^	Porter *et al*. criteria^26^	MSKCC criteria^14^	CARTOX criteria^12^
Grade 1	Symptoms are not life-threatening and require symptomatic treatment only, e.g. fever, nausea, fatigue, headache, myalgia, malaise	Mild reaction: treated with supportive care such as antipyretics, antiemetic	Mild symptoms, requiring observation or symptomatic management only (e.g. antipyretics, antiemetic, pain medications, etc.)	Temperature >38°C (fever Grade 1 organ toxicity
Grade 2	Symptoms require and respond to moderate intervention. Oxygen requirement <40% or hypotension responsive to fluids or low-dose pressor or Grade 2 organ toxicity	Moderate reaction: some signs of organ dysfunction (e.g. Grade 2 creatinine or Grade 3 LFTs) related to CRS and not attributable to any other condition. Hospitalization for management of CRS-related symptoms, including fevers with associated neutropenia, need for IV therapies (not including fluid resuscitation for hypotension)	Hypotension requiring any vasopressors <24 h, or Hypoxia or dyspnea requiring supplemental oxygen <40% (up to 6L NC)	Hypotension responding to IV fluids or low-dose vasopressors, hypoxia requiring FiO2 <40%, Grade 2 organ toxicity
Grade 3	Symptoms require and respond to aggressive intervention. Oxygen requirement ≥40% or hypotension requiring high-dose or multiple pressor or Grade 3 organ toxicity or Grade 4 transaminitis	More severe reaction: hospitalization required for management of symptoms related to organ dysfunction, including Grade 4 LFTs or Grade 3 creatinine related to CRS and not attributable to any other conditions; this excludes management of fever or myalgia; includes hypotension treated with intravenous fluids (defined as multiple fluid boluses for blood pressure support) or low-dose vasopressors, coagulopathy requiring fresh frozen plasma or cryoprecipitate, or fibrinogen concentrate, and hypoxia requiring supplemental oxygen (nasal cannula oxygen, high-flow oxygen, CPAP, or BiPAP). Patients admitted for management of suspected infection due to fevers and/or neutropenia may have Grade 2 CRS	Hypotension requiring any vasopressors ≥24 h, or Hypoxia or dyspnea requiring supplemental oxygen ≥40%	Hypotension needing high-dose or multiple vasopressors, hypoxia requiring FiO2 ≥40%, Grade 3 or Grade 4 transaminitis
Grade 4	Life-threatening symptoms. Requirements for ventilator support or grade 4 oxygen toxicity (excluding transaminitis)	Life-threatening complications such as hypotension requiring high-dose vasopressors, hypoxia requiring mechanical ventilation	Life-threatening symptoms Hypotension refractory to high-dose vasopressors *Hypoxia or dyspnea requiring mechanical ventilation	Life-threatening hypotension, Needing ventilator support, Grade 4 organ toxicity except for Grade 4 transaminitis

Lee *et al.* 2019^23^. Table is adapted from above publication.

Davila *et al*. also defined the severity of CRS based on cytokine levels and clinical features^16^. Under their criteria, severe CRS was characterized by fever greater than or less than 38°C for at least 3 consecutive days, two serum cytokines elevated at 75-fold over baseline, or one serum cytokine elevated 250-fold over baseline, and one clinical sign of severe toxicity. Severe toxicity could be in the form of hypotension requiring at least one intravenous vasoactive pressor or hypoxia (PO2 <90%) or neurologic disorders including mental status changes, obtundation, and seizures.

## Pathophysiology of CAR T-cell associated neurotoxicity

**Figure 1:**
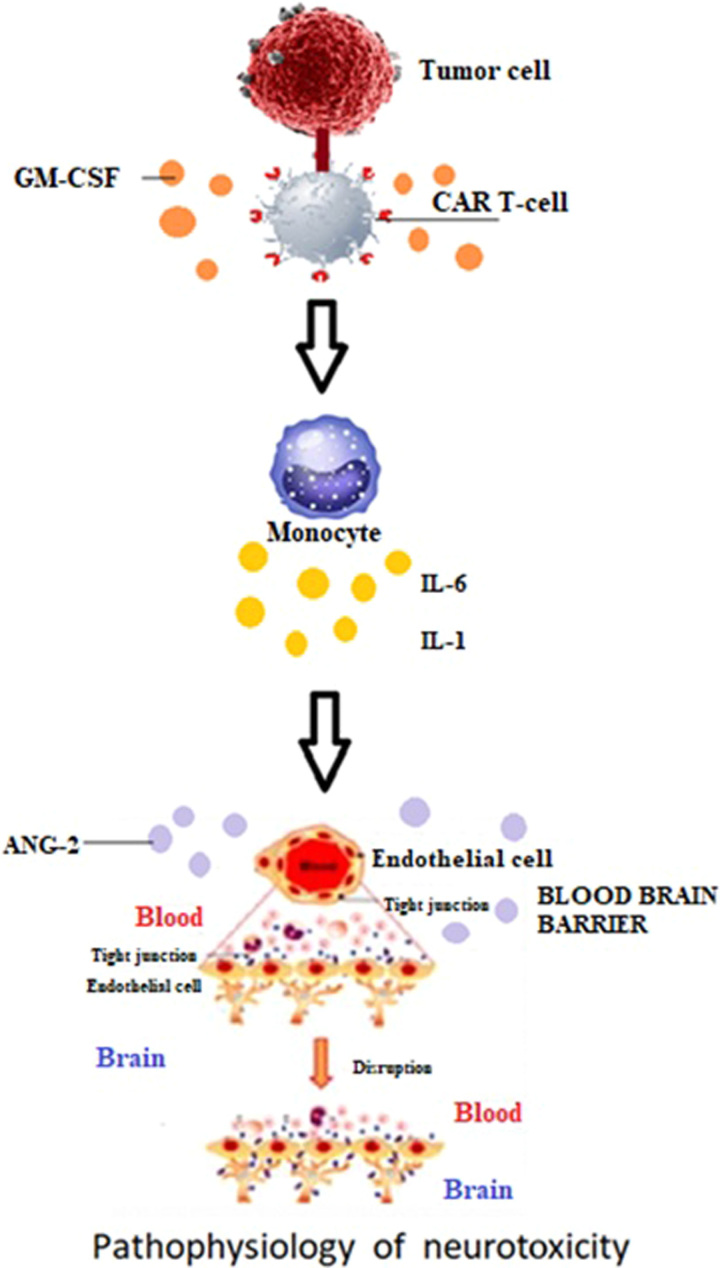
Pathophysiology underlying neurotoxicity in CAR T-cell therapy.

The pathophysiology of neurotoxicity and that of CRS has not been completely understood as of yet; however, certain mechanisms have been worked upon. The pathogenesis underlying neurological risk profile in CAR T-cell patients is demonstrated in Figure 1. In about 90% of patients, the onset of neurotoxicity occurs with CRS or after its resolution, the neurotoxicity that occurs without manifestation of CRS is mild or of grade 1. The mechanism of CRS leads to the activation of immune cells, that is monocytes and macrophages. The activated macrophages secrete large amounts cytokines including IL-6, IL-1, IL-10 inducible nitric oxide synthase (iNOS), and other mediators for inflammation. In a study of leukemic mice, monocytes were the main source of IL-1 and IL-6 during CRS, and inhibition of the IL-6 receptor (IL6R) with tocilizumab prevented CRS but did not affect neurotoxicity. Blocking IL1 with the IL1 receptor (IL1R) antagonist known as anakinra, prevented both CRS and neurotoxicity^27^.

Initially, neurotoxicity was linked to direct parenchymal CAR T-cell toxicity, however, new studies suggest that the dysfunction of the blood-brain barrier (BBB) is the main culprit^28^. Autopsy studies in patients who developed severe CRS along with high-grade neurotoxicity that progressed to fatal cerebral edema support dysfunctional BBB as well^29^. Another case report of a patient who developed fatal cerebral edema, showed evidence of BBB disruption^30^. BBB dysfunction has been associated with high levels of TNF-α, IL-6, and IL-1^31,32^. IL-6 has been proven to disrupt the endothelium of the BBB in vitro due to the low expression of intracellular tight junction molecules^33^. Furthermore, severe neurotoxicity has been linked with elevated levels of IL-15 and granulocyte-macrophage colony-stimulating factor (GM-CSF)^34^. It was suggested that the interaction of CAR T-cells with the tumor causes CAR T-cells to produce GM-CSF^35^, which acts as a bridge between the specific immune activity of the CAR T-cells and the off-target inflammatory cascade initiated by immune cells, which leads to myeloid cells to expand synthesis of other inflammatory chemokine and cytokines, including monocyte chemokine protein-1 (MCP-1), IL-1, and IL-6, and others.

In addition to this, a relatively higher level of angiopoietin 2 (ANG2) has been linked with severe neurotoxicity^36,37^. Angiopoietin 2 (ANG2) is secreted upon activation of endothelial cells by inflammatory cytokines and binds to the TIE2 receptor, which is present on the endothelial cell causing increased vascular permeability^38^. Angiopoietin 1 is a protein that is produced by perivascular cells, which surrounds the BBB, can be produced by platelets as well, and is usually found bound to the TIE2 receptor. Patients with high-grade neurotoxicity exhibited an increased ratio of ANG2 to angiopoietin 1 (ANG1). The earlier rise in ANG2 levels in the first 24 h following CAR T-cell therapy was associated with a higher risk of developing high-grade neurotoxicity, suggesting that endothelial activation precedes the development of clinical toxicity. Severe neurotoxicity was also linked with higher levels of von Willebrand factor (vWF), a blood glycoprotein involved in hemostasis, and IL-8 also known as a neutrophil chemotactic factor, both of which are stored in the same weibel-palade bodies, which are small storage granules located in endothelial cells as ANG2^28^. Endothelial activation by cytokines and inflammation following the CAR T-cell therapy causes the release of ANG2 and high molecular weight vWF, which results in increased vascular permeability and coagulopathy^28^. High-grade neurotoxicity is also linked with a higher concentration of biomarkers of diffuse intravascular coagulation with decreased levels of fibrinogen before the manifestation of neurologic signs or symptoms^28^.

## Clinical manifestations of CAR T-cell associated neurotoxicity

**Figure 2:**
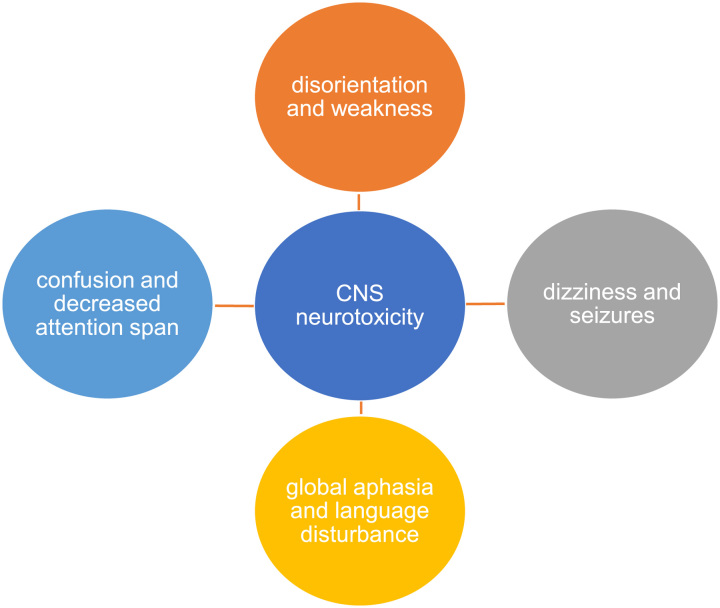
Clinical manifestations of CAR T-cell associated neurotoxicity.

The most frequent and severe toxicity of the CAR T-cell therapy is neurotoxicity, also called as CAR T-cell–related encephalopathy syndrome and immune effector cell-associated neurologic toxicity syndrome (ICANS)^39^. It is clinically presented with delirium, seizures, dizziness, decreased attention span, disorientation, ataxia, weakness, and sometimes headache as illustrated in Figure 2. It may gradually progress to confusion, difficulty in speaking, and global aphasia after expressive aphasia in severe cases^16,40,41^. Neurotoxicity greater than grade 2 is severe and is presented with motor weakness, incontinence, mental obtundation, and increased intracranial pressure, which causes papilledema and cerebral edema^12^. In addition to that, electroencephalography (EEG) detects the encephalopathy in patients presenting with neurotoxicity^28,42^. Severe neurotoxicity shows abnormal findings on MRI, which includes micro-hemorrhages, white matter changes, and leptomeningeal enhancement^28,43^. Severe ICANS often develop in patients with severe CRS, with a higher pretreatment tumor burden, younger age, and in patients with pre-existing neurological conditions^28^. Other than ICANS, CAR T neurotoxicity also include movement disorders, personality and cognitive changes as well as low incidence of peripheral neuropathies.

Relative frequencies of CRS occurrence is 3–71% as opposed to ICANS (0–56%). Generally, there is a lower rate of neurotoxicity observed in 4-1BB compared to CD28 co-stimulatory CD19 CAR T-cells. According to a study conducted in 2017 with CAR T-cells containing a 4-1BB co-stimulatory domain^28^, the median time for the presentation of neurotoxicity was reported to be 4 days after the CAR T-cell infusion. Around 40% of the patients had 1 and greater than 1 neurological toxicity whereas only 5% developed neurotoxicity greater than or equal to 4 whereas in the phase 2 ZUMA 1 trial with the CD28 co-stimulatory domain, the median duration of onset was observed to be 5 days and lasted 2–4 days while around 28% had neurotoxicity greater than or equal to grade 3^44^.

## Diagnosis of CAR T-cell-associated neurotoxicity

One of the most important findings for the detection of neurotoxicity is the change of the cerebrospinal fluid (CSF) composition. Neurotoxicity shows an increase in the serum protein levels along with an increase in the white blood cell count^28,45^. However, these markers are not specific as they can also be used to detect other dysfunctions^28,45,46^. Other serum biomarkers include C-reactive protein (CRP) and ferritin, which upon the administration of CAR T-cells rise above their normal levels. The peak concentration of ferritin varies with the low-grade and high-grade neurotoxicity whereas the peak CRP concentration does not depend on the severity^10^. Other laboratory findings include lactate dehydrogenase, coagulation assays, metabolites, and electrolyte levels^47^.

The physical examination includes neurological assessment, monitoring oxygen saturation, blood pressure, and temperature^47^. The neuroimaging includes brain MRI, which shows patchy T2 hyperintensities in the white matter, and symmetric T2 hyperintensities in the thalami in case of neurotoxicity^45,48^. The neurological injury can also be detected by the identification of global cerebral edema on imaging. In addition to MRI, a head computed tomography (CT) scan is performed, and according to a clinical trial^10^ only grade 4 neurotoxicity patients developed subdural hematoma almost 2 weeks after the onset of neurological symptoms, the rest showed normal CT imaging. Furthermore, electroencephalography which is more critical than MRI and CT scans detects the high-grade neurotoxicity characterized by the periodic or rhythmic EEG patterns on the ictal-interictal continuum^10^. The seizures associated with neurotoxicity are also detected by using EEG^49^. Along with that, to rule out papilledema, EEG and fundoscopic examination is performed in all cases of neurotoxicity^12^.

## Management and treatment of CAR T-cell-associated neurotoxicity

The management of neurotoxicity varies between different institutions and guidelines while the treatment of neurotoxicity depends on the severity which is determined by the grading criteria (Table 3) and is initiated by providing supportive care^42,50^. For patients with grade 1 ICANS, the platelet count and sodium levels are frequently monitored in addition to frequent neurological assessment, and corticosteroids are not administered unlike in patients presenting with symptoms showing grade 2 ICANS. The most common first-line corticosteroid used is dexamethasone as it is reported to penetrate the central nervous system well. Along with that, according to a report in 2018^12^, methylprednisolone is given in case of severe ICANS such as grade 4 ICANS depending on the neuroinflammatory disorders. In grade 3 ICANS, patients are admitted in the intensive care unit and electroencephalography, CT, and MRI are performed from time to time in case of increased intracranial pressure.

**Table 3:** Grading of immune effector cell-associated neurologic toxicity syndrome (ICANS)^23^.

Signs/Symptoms	GRADE 1	GRADE 2	GRADE 3	GRADE 4
ICE scores	7–9	3–6	0–2	0
Impairment	Mild	Moderate	Severe	Patient in critical condition
Seizures	No	No	Any clinical seizure focal or generalized that resolves rapidly; or nonconvulsive seizures on EEG that resolve with Intervention.	Life-threatening prolonged seizure (>5 min); or Repetitive clinical or electrical seizures without return to baseline in between.
Motor weakness	No	No	No	Hemiparesis and paraparesis.
Raised intracranial pressure	No	No	Focal/local edema on Neuroimaging. Stage 1–2 papilledema	Diffuse cerebral edema on neuroimaging; decerebrate or decorticate posturing; or cranial nerve VI palsy or stage 3–5 papilledema or Cushing’s triad.
Level of consciousness	Awakens spontaneously	Awakens to voice	Tactile stimulus is needed to awaken	Repetitive tactile stimulus is needed to awaken/ coma

Lee *et al.* 2019^23^. Table is adapted from above publication with a formal consent taken from the corresponding author.

Grade 4 ICANS is detected when there are repetitive seizures and increased intracranial pressure and is treated with high doses of the two corticosteroids^51^. Furthermore, according to a 2016 study^52^, siltuximab, a chimeric monoclonal antibody can be used to manage CRS and neurotoxicity both by directly binding interleukin-6, preventing it from binding with the IL-6 receptors. If the neurotoxicity is associated with CRS, tocilizumab 8 mg/kg IV can also be used instead of siltuximab^12^.

### Grade 1 ICANS

Patients are managed by supportive care, which includes minimizing the aspiration risks and giving intravenous fluids for hydration. For patients who have a disability in swallowing food or medications are also fed intravenously. Along with that, for grade 1 ICANS, MRI is performed in patients with focal peripheral neurological deficits, lumbar puncture for diagnostic purposes, CT scan is performed where an MRI is not feasible and EEG is carried out for 30 min every day until the symptoms resolve. Medications that can lead to central nervous system depression are avoided and levetiracetam 750 mg is given every 12 h as antiseizure prophylaxis^10,49^. They also include phenobarbital, which is used for seizures due to neurotoxicity and is preferred after levetiracetam. Low doses of lorazepam are likely to be administered every 8 h for patients who appear disconcerted^12^.

### Grade 2 ICANS

The patients are provided with supportive care and 10 mg IV dexamethasone is given every 6 h or 1 mg/kg IV methylprednisolone is given every 12 h when ICANS is not associated with CRS^12^.

### Grade 3 ICANS

Along with the repetitive neuroimaging every 2–3 days, the patients are given corticosteroids mainly dexamethasone 10–20 mg every 6 h in addition to supportive care^42^. If stage 1–2 papilledema is detected due to increased intracranial pressure in grade 3 ICANS, 1000 comparison of CRS grading using CTCAE versions 4.03 and 5.0 mg acetazolamide is administered intravenously, which is then followed by 250–1000 mg IV every 12 h^12^.

### Grade 4 ICANS

High-dose corticosteroids are administered until the neurotoxicity reaches grade 1 ICANS and then it is tapered. Repetitive neurological consultation and neuroimaging are also performed in addition to providing supportive care. Patients with grade 4 ICANS are often monitored in the ICU and mechanical ventilation is also provided to protect the airway as it is a severe condition^12,42^. Stages 3, 4, and 5 papilledema may be detected in patients with grade 4 ICANS. For treatment, high-dose corticosteroids are administered along with the hyperventilation and a 30° elevation of the head of the patient’s bed. In addition to that, hyperosmolar therapy with either mannitol (20 g/dl solution) or hypertonic saline (3% or 23.4%) can be administered^12^.

Some patients may develop nonconvulsive status epilepticus or convulsive status epilepticus regardless of the grade. For the management of the nonconvulsive status epilepticus, benzodiazepine^42^, which includes lorazepam, and antiepileptic, which includes levetiracetam are administered and maintenance doses for both are given even after the resolution of seizures. If the seizures persist, the patient is to be monitored in the intensive care unit along with the intravenous administration of phenobarbital 60 mg. Similar treatment is followed in the case of the convulsive status epilepticus except that the dosage of lorazepam is increased and there is a constant electroencephalogram monitoring^12^.

## Conclusion

CRS and neurotoxicity have a close association due to elevation in certain cytokines common in both types of toxicities. However, neurotoxicity is known to be caused by a compromised BBB and endothelial activation. The authors would like to emphasize that both have distinct pathophysiology that led to immune pathways that may be common. The CNS pathway coincides with the immune dysregulation of CRS. The severity of CRS is determined by the elevation of IL-6, IFN-Y, and soluble IL2Ra serum markers, which formulates the common pathway for CNS neurotoxicity. Management depends on the severity of the clinical symptoms determined by the grading criteria.

## Ethical approval

Ethical approval was waived by the institutional review board because of the study protocol as narrative review, which does not involve human subjects.

## Consent

Informed consent was not required for this review.

## Sources of funding

None to declare.

## Author contribution

F.Y.: conception of the study, major drafting of the work, final approval, and agreeing to the accuracy of the work; S.M.I.S.: conception of the study, major drafting of the work, final approval, and agreeing to the accuracy of the work; A.R.: conception of the study, major drafting of the work, final approval, and agreeing to the accuracy of the work; S.K.: help in design of the study, drafting of the work, final approval, and agreeing to the accuracy of the work; I.S.S.: help in design of the study, drafting of the work, final approval, and agreeing to the accuracy of the work; J.U.: help in design of the study, drafting of the work, final approval, and agreeing to the accuracy of the work; F.K.: help in design of the study, drafting of the work, final approval, and agreeing to the accuracy of the work; C.A.H.: help in design of the study, drafting of the work, final approval, and agreeing to the accuracy of the work; N.R.: help in design of the study, drafting of the work, final approval, and agreeing to the accuracy of the work; M.S.: help in design of the study, drafting of the work, final approval, and agreeing to the accuracy of the work; S.K.: help in design of the study, drafting of the work, final approval, and agreeing to the accuracy of the work; F.N.U.S.: help in design of the study, drafting of the work, final approval, and agreeing to the accuracy of the work; D.M.: help in design of the study, drafting of the work, final approval, and agreeing to the accuracy of the work. D.K.: help in design of the study, drafting of the work, final approval, and agreeing to the accuracy of the work; M.S.A.: help in design of the study, drafting of the work, final approval, and agreeing to the accuracy of the work.

## Conflicts of interest disclosures

The authors declare that they have no financial conflict of interest with regard to the content of this report.

## Research registration unique identifying number (UIN)

1. Name of the registry: not applicable.
2. Unique identifying number or registration ID: not applicable.
3. Hyperlink to your specific registration (must be publicly accessible and will be checked): not applicable.

## Guarantor

Muhammad Sohaib Asghar.

## Data availability statement

Used existing datasets publically available. No new datasets generated.

## Provenance and peer review

Not commissioned, externally peer-reviewed.
